# Perovskite/c-Si tandem solar cell with inverted nanopyramids: realizing high efficiency by controllable light trapping

**DOI:** 10.1038/srep16504

**Published:** 2015-11-13

**Authors:** Dai Shi, Yang Zeng, Wenzhong Shen

**Affiliations:** 1 Institute of Solar Energy, and Key Laboratory of Artificial Structures and Quantum Control (Ministry of Education), Department of Physics and Astronomy, Shanghai Jiao Tong University, Shanghai 200240, People’s Republic of China; 2 Collaborative Innovation Center of Advanced Microstructures, Nanjing 210093, People’s Republic of China

## Abstract

Perovskite/c-Si tandem solar cells (TSCs) have become a promising candidate in recent years for achieving efficiency over 30%. Although general analysis has shown very high upper limits for such TSCs, it remains largely unclear what specific optical structures could best approach these limits. Here we propose the combination of perovskite/c-Si tandem structure with inverted nanopyramid morphology as a practical way of achieving efficiency above 31% based on realistic solar cell parameters. By full-field simulation, we have shown that an ultra-low surface reflectance can be achieved by tuning the pyramid geometry within the range of experimental feasibility. More importantly, we have demonstrated that the index-guided modes can be excited within the top cell layer by introducing a TCO interlayer that prevents coupling of guided light energy into the bottom cell. This light trapping scheme has shown superior performance over the Bragg stack intermediate reflector utilized in previous micropyramid-based TSCs. Finally, by controlling the coupling between the top and bottom cell through the thickness of the interlayer, current generation within the tandem can be optimized for both two- and four-terminal configurations, yielding efficiencies of 31.9% and 32.0%, respectively. These results have provided useful guidelines for the fabrication of perovskite/c-Si TSCs.

Crystalline silicon (c-Si) solar cells have been dominating the photovoltaic market for years due to its high efficiency and mature industrialization. However, with its efficiency record approaching the Shockley-Queisser (S-Q) limit, further improvement becomes increasingly difficult. One potential solution is to introduce a wider-bandgap top cell and form a dual-junction tandem solar cell (TSC). In principle, such silicon-based TSC is able to selectively harvest different parts of the solar spectrum and surpass the 29.4% S-Q one junction limit. This notion has attracted great new interest following the recent discovery of a prominent candidate for the top cell material, namely the organometallic halide perovskite. Perovskite solar cells are of great photovoltaic potential with confirmed power conversion efficiency of 19.3% (for methylammonium lead iodide (CH_3_NH_3_PbI_3_))^1^ and 20.1% (for formamidinium lead iodide (FAPbI3))^2^. A typical CH_3_NH_3_PbI_3_ perovskite solar cell has high absorption coefficient^3^ and sharp absorption edge^4^, with a relatively large and tunable bandgap^5^, which makes it an ideal candidate to absorb the high-energy part of sunlight as a top cell.

To date, a few pioneering works have thoroughly assessed the efficiency upper limits of perovskite/c-Si TSC from a generalized point of view^6^,^7^,^8^. However, these investigations often overlook the specific structure of the TSC, and are based on simple assumptions including zero surface reflection, perfect intermediate reflector, and Lambertian light trapping in the top cell, etc. It remains unclear what kind of optical structure can fulfill all these criteria. Some other works have studied the performance of the perovskite/c-Si TSC with a micro-scale pyramidal surface the dimension of which is large enough to be treated by ray optics approximation^9^. Nevertheless, these micro-scale structures generally do not provide index-guided light trapping in the top cell, thus a complex Bragg stack intermediate reflector must be used to enhance the selective absorption of the tandem and maximize the efficiency^9^. It should be noted that one of the easiest ways of achieving selective absorption enhancement in a thin film solar cell (like a perovskite top cell) is to incorporate the incident light into the wave guided modes of the cell to increase its path length^10^,^11^,^12^,^13^, which could only be enabled by sub-micron periodicity. For this purpose, one possible choice is the inverted nanopyramids (with dimensions of 200 nm to 800 nm), which have recently been shown to provide excellent antireflection and light trapping for thin film c-Si solar cells^14^,^15^,^16^. Compared to other nanostructures like nanowires or nanopores, such inverted nanopyramids have smoother surface and lower surface area enhancement ratio^14^, which are beneficial for better conformal surface coverage in the perovskite/c-Si TSC application^15^. More importantly, the geometric parameters of the nanopyramids are tunable with fabrication conditions^16^,^17^, allowing for optimization of the overall tandem performance.

In this work, we have thoroughly studied the combination of perovskite/c-Si TSC with the inverted nanopyramid surface morphology by means of full-field simulation, and demonstrated its potential in realizing high efficiency by controllable index-guided light trapping in the top cell. In Section A of the discussion, we have first optimized the geometric parameters of the inverted nanopyramids which result in an overall surface reflectance as low as 2%. Then in Section B, we have revealed the physical mechanism that leads to the absence of light trapping in the top cell when the two cells are in direct contact. We have demonstrated the feasibility of restoring and controlling index-guided light trapping in the top cell by introducing a transparent conductive oxide interlayer (TCO IL), and shown its superiority over the traditional Bragg stack intermediate reflector. In Section C, we have demonstrated that the inverted nanopyramid tandem cell efficiency can be as high as 32% based on experimental cell parameters, and may reach over 35% with the development of perovskite material. These findings can serve as a practical guidance for the fabrication of high-efficiency perovskite/c-Si TSCs.

## Methods

**Figure 1:**
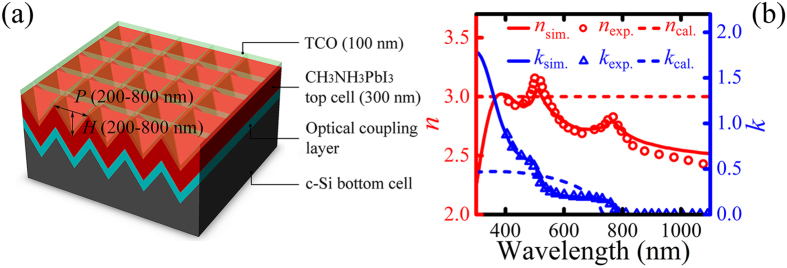
(**a**) Schematic 3D drawing of perovskite/c-Si TSC with inverted nanopyramid structure. The parameters *H* and *P* denote the pyramid height and its period, respectively. (**b**) Refractive index used in our simulation (*n*_sim_. and *k*_sim_.) and obtained by fitting experimental measurements (*n*_exp_. and *k*_exp_.), compared to the direct-bandgap fitting model (*n*_cal_. and *k*_cal_.).

Figure 1(a) shows the schematic drawing of the inverted nanopyramid perovskite-on-silicon TSC. The compositions that conduct an optical effect in the tandem structure consist of a 100-nm-thick top TCO contact (the hole collecting electrode), a 300-nm-thick CH_3_NH_3_PbI_3_ top cell, an infinitely-thick c-Si bottom cell, and an intermediate optical coupling layer the nature of which will be specified in the discussion (either none, a Bragg stack, or a TCO IL). Besides the aforementioned optical structures, real cells should also contain several electrical compositions that enable the transfer and collection of generated charges, which are: a 2,2′,7,7′-tetrakis(N,N-di-*p*-methoxyphenylamine)-9,9′-spirobifluorene (spiro-MeOTAD) layer and a compact TiO_2_ layer that sandwich the perovskite and act as the hole and electron transport material, respectively, and a metal (gold or silver) back contact under the silicon wafer^18^. Note that the hole transport layer is not an indispensable component for the perovskite cell^19^ and that TiO_2_ exhibits a refractive index (*n*) comparable with CH_3_NH_3_PbI_3_^20^ and marginal absorption under the AM1.5G spectrum^21^, so these electrical structures are eliminated from our optical model for generality. Both the top cell and the bottom cell possess an inverted nanopyramid feature defined by the pyramid height *H* and its period *P*, with values chosen in accordance with experimental observations.

To assess the tandem cell efficiency, we first perform finite-difference time-domain (FDTD) simulations to acquire the precise optical response of the cell. The incident light is set to be a plane wave propagating in the minus-z direction with a wavelength ranging from 300 nm to 1100 nm, covering the major AM1.5G solar spectrum. Periodic boundary conditions are used in the x-y directions and perfectly matched layer conditions are used in the z direction to simulate an infinite-area cell. The reflectance *R*(*λ*) normalized to the incident light power is obtained through the frequency-domain transmission monitor positioned at the top of the simulation region parallel to the cell surface. The absorptance of a specific layer (including top TCO, perovskite, or TCO IL, denoted by the form of *P*_abs_^(layer)^(*λ*)) can be obtained by the Analysis group “pabs” in the FDTD package.

To take into account the realistic absorption characteristics of the actual CH_3_NH_3_PbI_3_ material, we have taken the complex refractive indices (*n*, *k*) of the perovskite top cell from latest literature^4^,^22^ (shown in Fig. 1(b) by solid lines), instead of the direct-bandgap fitting model used in previous works^9^ that shows distinct differences (shown in Fig. 1(b) by dashed lines). For the same reason the bandgap of the top cell material is chosen as 1.55 eV instead of a wider one of 1.70 eV^9^ (note that 1.75 eV is the ideal bandgap for the top cell of a c-Si based TSC^23^). For the first section of discussion (Section A), parasitic optical loss in the TCO front layer is not considered to provide a more general idea of the optical properties of the nanopyramid structure (*n*_TCO_ = 1.5, *k*_TCO_ = 0 in this case). Whereas a realistic Indium-Tin Oxide (ITO)^24^ material is used as the TCO layers when discussing light trapping for the top cell in Sections B and C, since the increase of top cell absorption is achieved at the expense of parasitic loss caused by the conductive optical coupling layer. All other optical parameters are taken from literature^25^, and for simplicity, other parasitic losses are not considered.

After acquiring the reflectance and the absorptance in the top layers, we calculate maximum efficiency for each sub cell and thus the tandem efficiency. This is done by assuming an IQE of unity in the simulated materials, so the short-circuit current density (*J*_SC_) then corresponds to the integrated photon flux of the AM1.5G solar spectrum with the respective absorptance. The top cell *J*_SC_^(top)^ is calculated by


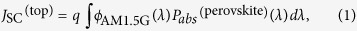


where *ϕ*_AM1.5G_(*λ*) is the incident photon flux, and *q* is the electron charge. The bottom cell *J*_SC_^(bottom)^ is calculated by subtracting the current loss caused by reflection (*J*_SC_^(*R*)^) and parasitic absorption (*J*_SC_^(TCO)^), as well as the top cell *J*_SC_^(top)^ from the AM1.5G full-spectrum current 43.5 mA/cm^2^


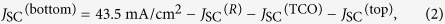


where


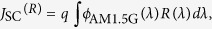


and


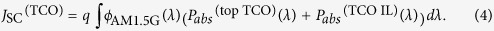


The open-circuit voltage (*V*_OC_) is calculated from the short-circuit current density *J*_SC_ by the one-diode equation


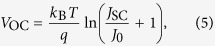


where *k*_B_ is the Boltzmann constant, and *T* is the room temperature 298 K. *J*_0_ in equation (5) is the diode saturation current density, which has been calculated from experimental results for both cells: for the top cell, *J*_0_^(top)^ = 1.76082 × 10^−17^ mA/cm^2^, derived from the illuminated *I*–*V* curve of the perovskite solar cell presented by Liu *et al.*^26^ with *V*_OC_ = 1.07 V and *J*_SC_ = 21.5 mA/cm^2^; while for the bottom cell, *J*_0_^(bottom)^ = 4.9 × 10^−11^ mA/cm^2^, derived from the world record c-Si solar cell reported by Zhao *et al*.^27^ with *V*_OC_ = 0.706 V and *J*_SC_ = 42.7 mA/cm^2^ 28. The fill factor *FF* is calculated using the well-established expression^29^


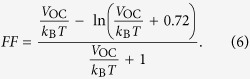


With the maximum output power per unit area from the respective cell calculated by


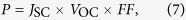


the overall efficiency of the simulated perovskite/c-Si TSC is finally obtained by


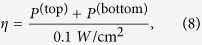


where *P*^(top)^ and *P*^(bottom)^ are top and bottom cell output power calculated from equation (7).

All simulations in this work are performed using a commercial software package [FDTD Solutions v8, Lumerical 2013], the validity of which has been proven by numerous works regarding nano-scale optoelectronic devices.

## Results and Discussion

With the model developed in the previous section, we are able to identify the performance of the perovskite/c-Si TSC of variant parameter values and structure details. In Section A, we first consider the case where there is no optical coupling layer between the top and bottom cells, and focus on the dependence of overall reflectance on the structural parameters of the inverted nanopyramids. We have investigated into the reflection and top cell absorption within a large parameter space by simultaneously varying the values of *P* and *H* (from 200 nm to 800 nm). The optimum realizable dimension parameters are then decided according to the simulation results. Then in Section B, we establish further discussion concerning the light trapping strategy of the top cell based on the previously determined *P* and *H*. We elucidate the difference on light trapping mechanism between a Bragg stack and a TCO IL in our tandem structure. At last, we calculate in Section C the tandem efficiency for two cell configurations: a mechanical stack of independently connected cells (four-terminal) and a monolithically integrated device (two-terminal).

## Perovskite/c-Si TSC with varying inverted nanopyramid dimensions

**Figure 2:**
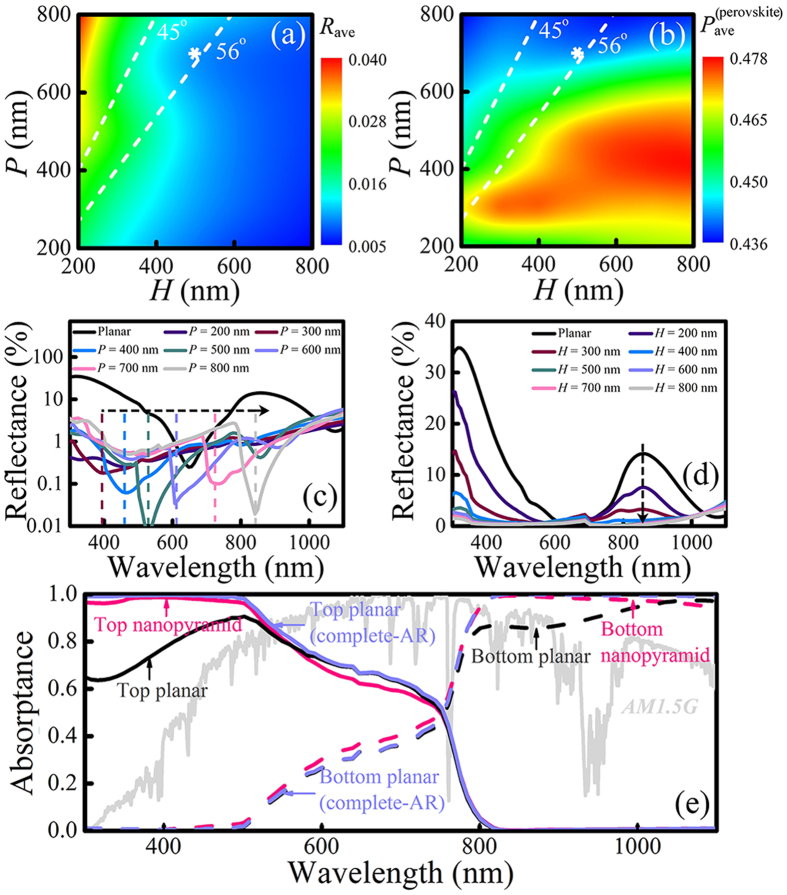
(**a**,**b**) Contour of AM1.5G-averaged reflection (*R*_ave_) and top cell absorption (*P*_ave_^(perovskite)^), respectively. White dashed lines mark the experimental realizable base angle limits. (**c,d**) Reflectance of inverted nanopyramid TSC with *H* fixed at 500 nm and with *P* fixed at 700 nm, respectively. Black dashed arrows indicate tendency of curves with increasing *P* or *H*. The colored dashed lines in (**c**) mark the local minimums of reflectance caused by Wood-Rayleigh anomaly. (**e**) Top and bottom cell absorptance for nanopyramid structured TSC with the optimum parameters (*H* = 500 nm/*P* = 700 nm) and the planar TSC. A normalized AM1.5G solar spectrum is overlaid in gray for reference.

As a general principle, the antireflection property determines the amount of sunlight that can be utilized in a solar cell and is thus a premise for achieving maximum efficiency, which makes it our first concern in designing the structure of the perovskite/c-Si TSC. In Fig. 2(a,b), the influence of the pyramid geometric parameters *P* and *H* on the AM1.5G-averaged reflection (*R*_ave_) of the tandem cell and the top cell absorption (*P*_ave_^(perovskite)^) is shown as color contours (note that in this section, the top and bottom cells contact directly with no intermediate optical coupling layer, and the top TCO layer conducts no parasitic absorption). It should be pointed out that the common fabrication techniques of silicon nanopyramids over large surface area only allow base angle *α* (*α* = arctan(*H*/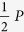)) in a limited range (from 45 to 56 degrees^17^,^30^, shown by the region between the two dashed lines) around the 54.74 degree of {111} facet. Nevertheless, here we explore a relatively larger parameter space in order to reveal the underlying physical mechanisms more clearly and make the conclusions compatible with future progress of nanopyramid fabrication. The two figures show obvious dependence of *R*_ave_ and *P*_ave_^(perovskite)^ on the inverted nanopyramid dimensions. In general, higher *H* leads to lower reflection and larger absorption of light in the top cell, which is mainly attributed to a more gradual change of refractive index from air to the cell bulk. Increased scattering of incident light by pyramids with larger height may be another factor that contributes to the reduced reflection, nevertheless further investigation remains to be developed. The prominent antireflection property of inverted nanopyramid structure is clearly demonstrated by the fact that a majority of the area in Fig. 2(a) has a *R*_ave_ lower than 2%, a promising value compared to other antireflection nanostructures^13^,^31^ or the conventional micrometer texture^9^. Although the choice of the geometric parameters indeed has a slight influence on the top cell absorption *P*_ave_^(perovskite)^, it can mostly be attributed to the change in total reflection as is revealed by referring to the same area in Fig. 2(a). Comparison of the two contours elucidates the marginal light trapping effect in this kind of preliminary inverted nanopyramid TSC structure. Similar conclusions have been drawn by other researchers for the cases of micropyramids as well^9^,^31^. The complete absence of light trapping in our nano-scale periodic structures may seem unexpected and will be explained later, however it allow us to focus only on reducing the overall reflection in this stage of optimization. Regarding this, we have chosen the optimum case with *H* = 500 nm and *P* = 700 nm to carry out further discussion, which is marked by the white asterisks. It should be noted that apart from the present experimental constrains, higher *α* is encouraged to obtain lower surface reflection and hence more absorption of incident light.

To further elucidate the antireflection mechanism, we have provided the wavelength dependence of overall reflection on the parameter *P* and *H*, respectively, at the previously chosen point *H* = 500 nm and *P* = 700 nm. Fig. 2(c,d) show the surface reflectance of varying *P* (from 200 nm to 800 nm) at fixed *H* (500 nm) and varying *H* (from 200 nm to 800 nm) at fixed *P* (700 nm), respectively. Reflectance of a planar TSC with TCO top contact of the same thickness and no optical interlayer is given for comparison. Both cases show dramatic reduction of reflectance compared to the planar cell (note that Fig. 2(c) has a logarithm y-axis). As a result, the *R*_ave_ diminishes from ~18% for the planar cell to less than 2% for the inverted nanopyramid ones. Black dashed arrows on the figures indicate tendency of curves with increasing *P* or *H*. Local minimums of reflectance in the visible-light region are marked by dashed lines in Fig. 2(c). While the minimum of the planar cell reflectance is determined by the destructive interference condition of the top TCO layer, the sharp drops of reflectance of the inverted nanopyramid ones are due to the Wood-Rayleigh (WR) anomaly effect caused by the periodic array^32^. The WR anomaly in inverted nanopyramid structure allows the regulation of lowest reflectivity at wished wavelength, which could be taken into consideration in the cell fabrication. While *P* has an influence on the position of reflectance minimum, *H* mainly influences the magnitude of reflectance. From Fig. 2(d) we see that planar cell suffers from significant reflection loss at short- (<500 nm) and long- (~850 nm) wavelength, where the destructive interference condition is not met. This status could be distinctly improved by introducing the inverted nanopyramid morphology. More importantly, we can see that the reflectance drops rapidly with increasing *H*, almost saturating for *H* values larger than 500 nm. Such weak dependence on excessive nanopyramid height may facilitate the design as well as the fabrication of the TSC in practice.

In Fig. 2(e), the absorptance of the top and bottom cell under the optimized pyramid parameters *H* = 500 nm/ *P* = 700 nm is shown as pink lines. Reduced reflection results in enhancement in absorption in both top and bottom cells compared to the planar TSC case (black lines), especially at the wavelengths where the reflection is remarkably diminished (<500 nm and ~850 nm). With the inverted nanopyramid structure preventing light from reflecting, top cell absorptance at short-wavelength almost reaches 100%. However, the absorptance of the top cell at mid-wavelength (600 to 800 nm) remains at a relatively low level. Provided that CH_3_NH_3_PbI_3_ has a sharp absorbing edge at *λ* = 800 nm, it can be concluded that considerable amount of light has been transmitted to and absorbed by the bottom cell. If we exclude the difference in antireflection by dividing the absorptance of the planar TSC by (1 − *R*(*λ*)), with *R*(*λ*) being the simulated reflectance from the cell, the resulted curves (lilac lines denote by “complete-AR”) represent a baseline of light trapping in the planar structure. Apparently, little difference could be seen between the inverted nanopyramid case and the planar complete-AR one, which again illustrates that the nanopyramid texture makes no contribution to the light trapping of the top cell. Similar phenomenon occurs if we apply the same notion (complete-AR) onto the results of Li *et al.* for a-Si/c-Si TSC^15^. Besides, it can be seen that the nanopyramid texture even promotes the coupling of light from the top cell to the bottom cell for certain mid-wavelengths, embodied by a slight decrease of top cell absorption below the planar baseline. Thus, in order to approach an ideal selective absorption in the top and bottom cells, effective light trapping strategy in the top cell should be established.

## Light trapping strategy: Index guiding enabled by TCO IL

The most common way of achieving light trapping in thin-film solar cells is to utilize the wave-guiding property of their component high-index material^11^. Such high-index material tend to support guided modes in which the electro-magnetic fields are localized in the vicinity of the cell, and the coupling of incident light into these modes will result in a significant enhancement of absorption. Two factors are crucial for realizing index-guided light trapping: first, the structure must provide phase-matching for the incident light to couple to the guided modes; second, a sufficiently large contrast in dielectric constant must exist to prevent the evanescent waves in the vertical direction from coupling to external material and causing a leakage. In our preliminary TSC structure, the first condition has been satisfied by the existence of periodic nanopyramids, however the second condition is not met due to the direct contact between the top and bottom cells. The similar dielectric constants of the top and bottom cells allow the confined light to propagate away into the tandem cell bulk, invalidating the index-guided light trapping. As an alternative, Bragg stacks have often been used in TSCs to enhance the absorption of the top cell^33^,^34^. A Bragg stack, which is essentially a 1-D photonic crystal, can act as a wavelength-selective intermediate reflector which reflects short-wavelength light back into the top cell and enhances its absorption. However, such multi-layer structure significantly augments the complexity of the fabrication process and affects the longitudinal electrical conductivity, therefore is of limited practical use in both two- and four-terminal tandem devices.

**Figure 3:**
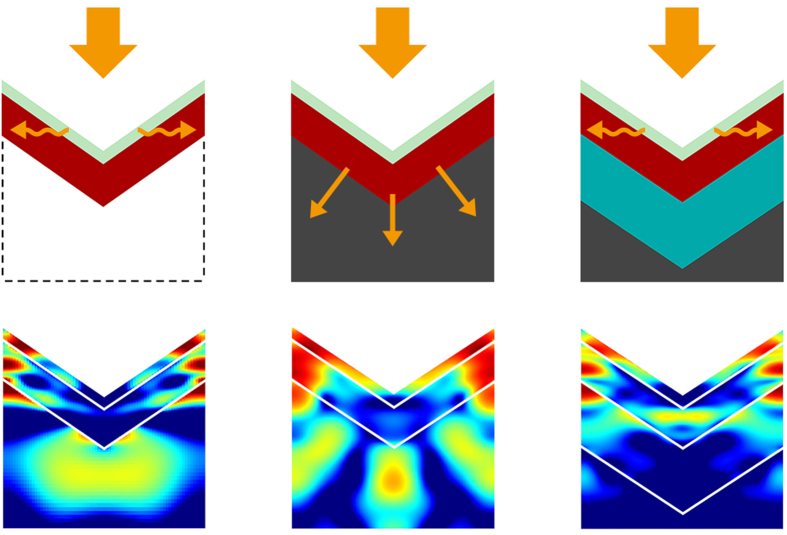
Sketch (top) and light distribution profiles of *λ* = 650 nm (bottom) for three scenarios: freestanding perovskite top cell in air (left), perovskite/c-Si TSC without IL (middle), and perovskite/c-Si TSC with 500-nm-thick ITO IL (right). Orange arrows indicate the propagation path of light. The color scales at the bottom are normalized separately for each case.

Here we investigate the possibility of restoring index-guided light trapping in the top cell by introducing a single TCO separation layer between the two cells. The distinctly smaller refractive index of TCO can provide a spatial detachment for the confined modes and enable wave-guiding by the perovskite material, while its excellent conductivity guarantees electrical contact in both two- or four-terminal tandem structures. To elucidate the feasibility of such method, we have simulated three different scenarios as are shown in Fig. 3: a freestanding perovskite top cell in air (left), a perovskite/c-Si TSC without IL (middle), and a perovskite/c-Si TSC with 500-nm-thick ITO IL (right). All three cases have the same inverted nanopyramid parameters *H* = 500 nm/*P* = 700 nm, and their light distribution profiles for mid-wavelength light *λ* = 650 nm are shown below by color (each normalized separately to best show its characteristic). For the freestanding perovskite top cell (left), we can clearly see that most light energy is confined within the vicinity of the cell material, showing effective excitation of the horizontal guided modes. Although a small portion of the electric field extend further below the pyramid vertex, the absence of any high-index material prevents the coupled light from escaping. As a result, a *J*_SC_ of 25.3 mA/cm^2^ is generated within the perovskite top cell, approaching the 27.3 mA/cm^2^ *J*_SC_ limit for complete conversion of solar radiation with a material bandgap of *E*_g_ = 1.55 eV, while a planar counterpart can only generate 19.6 mA/cm^2^ (complete-AR). By sharp contrast, when the c-Si bottom cell is placed directly below the top cell (middle), a strong leaky path is observed that effectively channels light into the c-Si bulk, leaving only a weak concentration of light energy in the top cell. Such leaky paths for the confined light may facilitate the overall antireflection performance, as has been reported for other resonant structures on a substrate^13^,^30^, however it greatly diminishes the absorption of the top cell from 25.3 mA/cm^2^ in the freestanding case to 19.2 mA/cm^2^ in the tandem structure. When we introduce an ITO IL between the top and bottom cells (right), pronounced index-guided modes can again be seen in the perovskite top cell, with a light distribution profile closely resembling that of the freestanding case. The suppression of the leaky channels of light leads to its confinement within the perovskite material, increasing the top cell *J*_SC_ by 18.7% from 19.2 mA/cm^2^ to 22.8 mA/cm^2^. Thus, it is evident that index-guided light trapping can be restored in the tandem cell with the help of an optical detaching layer. Finally, it is worth pointing out that the extent to which mid-wavelength light couples and leaks into the bottom cell can be controlled through the thickness of the intermediate TCO, a feature that we will use to achieve current-matching for two-terminal tandem devices.

**Figure 4:**
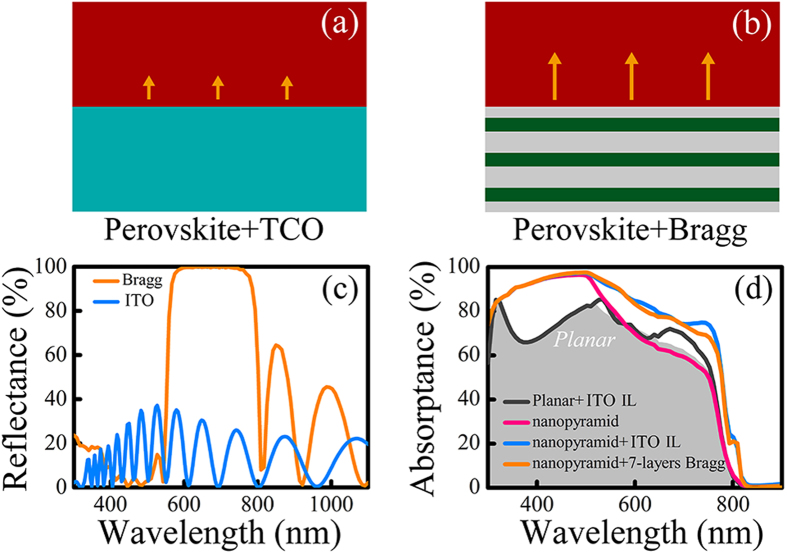
(**a**,**b**) Sketch for reflection at the interface of perovskite/TCO and perovskite/Bragg stack, respectively. (**c**) Interface reflectance for perovskite/ITO and perovskite/Bragg stack on silicon substrate. (**d**) Top cell absorptance for different cell structures: planar cell with ITO IL, nanopyramid without IL, nanopyramid with ITO IL, and nanopyramid with a 7-layer Bragg stack reflector. The absorption of the planar cell without any texture or light coupling interlayer is overlaid in gray for reference.

To further reveal the different mechanisms of light trapping enabled by a TCO IL and by a Bragg stack, we have simulated the reflection characteristics of the interfaces between perovskite/TCO and perovskite/Bragg stack, as illustrated in Fig. 4(a,b), respectively. Fig. 4(c) shows the wavelength-dependent reflectance for both cases on infinitely-thick c-Si substrate. The simulated Bragg stack is composed of alternating TiO_2_ (*n*_H_ = 2.4) and SiO_2_ (*n*_L_ = 1.46) layers with relative thickness fixed to maintain an equal optical length in each material^9^. Additional *λ*_0_/8 TiO_2_ layers are placed on both sides of the stack to maximize the transmittance of long-wavelength light^35^. By carefully choosing the parameters for the Bragg stack, its reflecting peak is located above the absorption edge of the CH_3_NH_3_PbI_3_ material (*λ* = 800 nm) to maximize the selective absorption of the tandem cell. As can be seen in Fig. 4(c), the perovskite/Bragg stack interface shows total reflection of light in a certain wavelength range, proving that it enhances the top cell absorption by giving the non-absorbed (mid-wavelength) light a second chance to pass through the top cell. Meanwhile, the perovskite/ITO interface shows a broadband low reflectivity, indicating a light trapping mechanism distinctively different from selective reflection. Such difference is more directly demonstrated in Fig. 4(d), where we have plotted the top cell absorptance for a planar TSC (gray shade), a planar TSC with ITO IL (dark gray line), an inverted nanopyramid TSC (pink line), an inverted nanopyramid TSC with ITO IL (blue line), and an inverted nanopyramid TSC with a Bragg stack of the same thickness (orange line), respectively. It should be noted that the slight difference of the top cell absorbing curves in Fig. 4(d) and Fig. 2(e) comes from the different dielectric constants used for the TCO layers in the two respective sections. ITO exhibits a refractive index of ~2.2 at wavelength of 300 nm, which satisfies the destructive interference condition for the 100 nm-thick top layer, resulting in an enhancement of absorption at ~300 nm. Compared to the planar TSC, the introduction of an ITO IL only brings about a slight increase in absorption in the mid-wavelength range (gray shade to dark gray line). This is due to the fact that the planar structure cannot provide the essential in-plane wave vector for normal incident light to couple to the guided modes of the perovskite slab, so the only light trapping achievable is by the weak reflection of mid-wavelength light on the perovskite/ITO interface. As a result, a small relative increase of only 3.8% in top cell *J*_SC_ is observed. However, for the inverted nanopyramid cases, the introduction of an ITO IL shows a significant enhancement in top cell absorption compared to the one with direct cell contact (pink line to blue line), leading to a relative increase of 18.7% in *J*_SC_. As has been discussed above, the excitation of index-guided modes is the dominant factor of light trapping in this case. It can also be seen that such index-guided light trapping even slightly out performs the optimized 7-layer Bragg stack, with a top cell *J*_SC_ comparison of 22.8 mA/cm^2^ to 22.4 mA/cm^2^. Finally, it is worth pointing out that the incomplete top cell absorption of short-wavelength light mainly results from the parasitic loss from the ITO top layer, which causes a *J*_SC_ loss of 0.5 mA/cm^2^.

## Tandem efficiency

**Figure 5:**
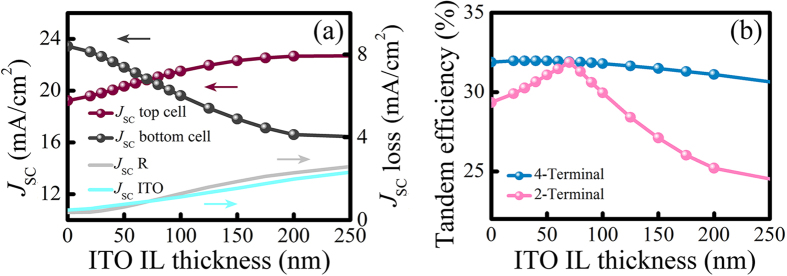
(**a**) Short-circuit current density of the respective sub cells for varying ITO IL thicknesses. The *J*_SC_ losses caused by tandem reflectance and parasitic ITO absorption are presented for reference. (**b**) Tandem efficiency for four-terminal and two-terminal TSCs, respectively.

So far we have optimized the inverted nanopyramid parameters to best suppress overall reflection, and established an index-guided light trapping strategy to maximize the selective absorption of the tandem device. In the following we will focus on the dependence of tandem efficiency on the thickness of the IL. As mentioned above, thicker ITO IL generally leads to more effective light trapping in the top cell, however usually at the expense of increases in reflectance and parasitic loss. In Fig. 5(a), we have plotted the dependence of top and bottom cell *J*_SC_ and the overall reflectance on ITO IL thickness. With increasing ITO IL thickness, the top cell *J*_SC_ rises rapidly from 19.2 mA/cm^2^ to a current-matching value of 20.8 mA/cm^2^ at an ITO thickness of 70 nm, and further saturates around 22 mA/cm^2^ for ITO ILs thicker than 200 nm. The *J*_SC_ of the bottom cell shows the inverse trend, dropping from 23.4 mA/cm^2^ without ITO IL to 16.6 mA/cm^2^ at an ITO IL thickness of 200 nm. The larger change in bottom cell *J*_SC_ is attributed to the increases in both reflectance and parasitic loss, with reflectance loss rising from 0.4 mA/cm^2^ to 2.3 mA/cm^2^ at 200 nm, and ITO parasitic loss rising from 0.5 mA/cm^2^ to 2.0 mA/cm^2^.

With these *J*_SC_ values, we can calculate the perovskite/c-Si TSC efficiency for two cell configurations: mechanical stack of independently connected cells (four-terminal) and monolithically integrated devices (two-terminal), as are shown in Fig. 5(b). For the four-terminal configuration, tandem efficiency remains at 31% to 32% for all ITO IL thicknesses, in contrast to the conclusions of previous theoretical investigations where the tandem efficiency is expected to increase monotonically with enhanced selective absorption^7^. This is due to the fact that in realistic cell structures, the trapping of short- and mid-wavelength light is inherently contradictory with the coupling requirement for maximizing the antireflection performance, especially by applying an IL that induces additional parasitic loss. Thus a trade-off must be made between selective absorption and overall losses. Specifically in this case, with the augmentation of losses for large ITO IL thicknesses, the maximum of four-terminal tandem efficiency is found at the ITO IL thickness of 30 nm, with a value of 32.0%. For the two-terminal configuration, current-matching condition clearly plays the most important role in determining the tandem efficiency. Insufficient or excessive absorption in the top cell both result in a dramatic lowering of the tandem efficiency, as the tandem current is determined by the lower one of the two sub cells. At the current-matching point (ITO thickness equals 70 nm), the top and bottom cells have equal *J*_SC_ of 20.9 mA/cm^2^, leading to a tandem efficiency of 31.9%, which falls only slightly short of the highest four-terminal efficiency of 32.0%. These numbers clearly suggest the feasibility of the two-terminal tandem structure over the four-terminal one when applied to a practical TSC.

Finally, we extend our calculations to include the highest achievable tandem efficiency with the future development of the perovskite top cell. Latest researches have already shown extraordinary 0.55 luminescence efficiency for the perovskite material^36^, much higher than the one used above (~10^−4^) extracted from state-of-the-art perovskite solar cell. Assuming a luminescence efficiency *ϕ* of 0.55, the lowest limit (radiation limit) of diode saturation current density *J*_0_ of a perovskite solar cell is calculated by


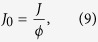


where


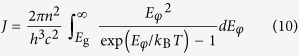


is the background blackbody flux at ambient temperature^37^, *E*_*φ*_ is the incident photon energy at wavelength *λ*: *E*_*φ*_(*λ*) = *hc*/*λ*, *E*_g_ = 1.55 eV is the bandgap of the CH_3_NH_3_PbI_3_ material, *n* is the refractive index of the ambient space, *h* is the planck constant, and *c* is the velocity of light. With the *J*_0_ calculated by equation (9), much higher tandem efficiencies can be obtained: 35.7% for the four-terminal TSC and 35.6% for the two-terminal TSC, showing significant potential of the perovskite/c-Si TSC structure.

In summary, we have studied the combination of perovskite/c-Si TSC structure with the inverted nanopyramid morphology as a practical way of achieving high-efficiency tandem device. We have found an optimized set of pyramid parameters *H* = 500 nm/*P* = 700 nm that suppresses the overall reflectance to as low as 2%, serving as a premise for high efficiency. Then by introducing a TCO IL, we have further shown that the index-guided light trapping can be restored in the top cell to significantly enhance the selective absorption of the tandem. This effect also provides us with the means to control the current generated in each sub cell through the thickness of the IL. As a result, tandem efficiencies of 32.0% and 31.9% have been demonstrated for four-terminal and two-terminal configurations, respectively, with the hope of reaching over 35% following future material development. These results have offered an applicable way of fabricating high-efficiency perovskite/c-Si TSCs.

## Additional Information

**How to cite this article**: Shi, D. *et al.* Perovskite/c-Si tandem solar cell with inverted nanopyramids: realizing high efficiency by controllable light trapping. *Sci. Rep.* **5**, 16504; doi: 10.1038/srep16504 (2015).
